# Diagnostic Accuracy of Multi-Parametric Magnetic Resonance Imaging for Tumor Staging of Bladder Cancer: Meta-Analysis

**DOI:** 10.3389/fonc.2019.00981

**Published:** 2019-10-04

**Authors:** Nieke Zhang, Xiaoyan Wang, Chunying Wang, Shuqiu Chen, Jianping Wu, Guangyuan Zhang, Weidong Zhu, Jing Liu, Bin Xu, Mulong Du, Ming Chen

**Affiliations:** ^1^Department of Urology, Affiliated Zhongda Hospital of Southeast University, Nanjing, China; ^2^Department of Nursing, Affiliated Zhongda Hospital of Southeast University, Nanjing, China; ^3^Jiangsu Key Laboratory of Cancer Biomarkers, Prevention and Treatment, Department of Environmental Genomics, Collaborative Innovation Center for Cancer Personalized Medicine, Nanjing Medical University, Nanjing, China; ^4^Department of Biostatistics, Center for Global Health, School of Public Health, Nanjing Medical University, Nanjing, China

**Keywords:** bladder cancer, multi-parametric magnetic resonance imaging, diagnosis, staging, meta-analysis

## Abstract

**Purpose:** Evaluate the diagnostic accuracy of multi-parametric magnetic resonance imaging (mp-MRI) for local staging of bladder cancer (BCa).

**Materials and Methods:** The databases of PubMed, Web of Science, Wanfang, and CNKI were searched for related literatures about BCa diagnosed by mp-MRI from January 1, 2000 to April 12, 2019. The strict inclusion and exclusion criteria were set up to extract records. The Quality Assessment of Diagnostic Accuracy Studies (QUADAS)-2 was used to evaluate quality of the candidate studies. The pooled sensitivity, specificity, positive likelihood ratio (+LR), negative likelihood ratio (−LR), and diagnostic odds ratio (DOR) were calculated to assess the diagnostic authenticity of mp-MRI. The summarized receiver operating characteristic (SROC) curve corresponding with the area under the curve (AUC) were analyzed to comprehensively evacuate the diagnostic value of mp-MRI.

**Results:** A total of 140 studies were retrieved by computer-based searching. After quality control, 4 studies with 259 patients were enrolled for meta-analysis. The pooled results showed 0.84 of sensitivity [95% confidence interval (CI) = 0.79-0.89], 0.91 of specificity (95% CI = 0.87–0.93), 8.24 of +LR (95% CI = 4.87–13.92), 0.18 of –LR (95% CI = 0.10–0.31), 49.42 of DOR (95% CI = 19.07–128.09), and 0.946 of AUC. The Spearman correlation analysis found no threshold effect (*p* = 0.684). A significant heterogeneity existed among 4 included studies with sensitivity (I^2^ = 65.7%), specificity (*I*^2^ = 60.0%) and diagnostic OR (*I*^2^ = 67.5%). The Begg's test (*p* = 0.497) and the egger's test (*p* = 0.337) found no publication bias.

**Conclusion:** mp-MRI acts a good diagnostic performance for bladder cancer. It is plausible that mpMRIs can be used as an important method for bladder cancer staging.

## Introduction

Bladder cancer is one of the most common cancers worldwide with nearly 550,000 new cases diagnosed in 2018 and around 200,000 patients die from this disease. Its incidence and prevalence have been increasing constantly in the past decade (1, 2). Treatment recommendations of the practice guidelines are based on the tumor and grade which is determined by the combination of clinical symptoms and imaging parameters (3).

Currently, the multi-parametric magnetic resonance imaging (mp-MRI) is widely used for bladder cancer diagnosis and staging. It consists of the conventional sequence [T2-weighted anatomic imaging (T2WI)] and functional MRI techniques [dynamic contrast-enhanced (DCE) imaging and diffusion-weighted imaging (DWI)] (4). Multi-parametric magnetic resonance imaging has the high sensitivity and specificity in diagnosing and staging the bladder cancer. In addition, it can provide more information about the morphology of bladder and the blood supply, so it is helpful for preoperative diagnosis of bladder cancer (4). In the recent decade, several studies have assessed the diagnosis value of mp-MRI in staging bladder cancer, however the sensitivity and specificity of each study were not in accordance.

This study is aimed to comprehensively evaluate the diagnostic ability of mp-MRI in staging bladder cancer and convincingly provide evidences for supporting the treatment in the clinic.

## Materials and Methods

### Searching Strategies

The computer search was performed among the databases of PubMed, Web of Science, Wanfang, and CNKI with the search terms including (“bladder cancer” OR “BCa” OR “bladder carcinoma” OR “urinary bladder neoplasms”) AND (“mp-MRI” OR “multi-parametric magnetic resonance imaging” OR “multi-parametric MRI” OR “MRI” OR “magnetic resonance imaging”) AND (“staging” OR “stage”) AND (“diagnosis” OR “diagnose”-). The studies were published from January 1, 2000 to April 12, 2019. Titles and abstracts were screened for relevance, full texts reviewed and the inclusion and exclusion criteria were applied to select the records.

### Inclusion Criteria

The inclusion criteria were set as follows: (a) Studies of mp-MRI for local staging of the bladder cancer in human (field strength ≥1.5 T); (b) Data was available to calculate the 2^*^2 contingency table; (c) Pathological results were used as the reference standard; (d) Staging the bladder cancer by mp-MRI included T2WI, DWI, and DCE-MRI; (e) Number of patients ≥ 10.

### Exclusion Criteria

The exclusion criteria were set as follows: (a) The study was a case report, editorial, review, systematic review, or commentary; (b) Patients were included in another study; (c) Stage of bladder cancer was judged by only one reader based on imaging results.

### Risk Bias Assessment of Included Studies

The quality of the included literature was assessed according to the QUADAS-2 tool recommended by the Cochrane collaboration web (5).

### Data Extraction

Reading the title and abstract was the first step toward excluding the obviously irrelevant records. The full texts were then screened according to the inclusion and exclusion criteria. Information extracted from the records included characteristics of records [first author, publication year, study period, country and language, number of reader, golden standard, research type, the true positive(TP), false positive(FP), false negative(FN), true negative(TN)], characteristics of patients [number of patients(male and female) and age], and characteristics of mp-MRI (manufacturer and model of the scanner, magnet strength, repetition time, echo time, slice thickness, slice gap, and flip angle of the T2WI, DWI, DCE).

### Statistical Method

We used the Meta-DiSc 1.4, Stata 14.0, and Review Manager (RevMan. Version 5.3) to analyze data. First, Spearman correlation analysis was used to test heterogeneity caused by threshold effect, if *P* > 0.05, means there is no heterogeneity, the fixed effect model is adopted; if *P* < 0.05, means heterogeneity exists, the random effects model is adopted. We merged the evaluation index by calculating pooled sensitivity (Sen), pooled specificity (Spe), pooled positive likelihood ratio (+LR), pooled negative likelihood ratio (-LR), and pooled diagnostic odds ratio (DOR); then also drew a summarized receiver operating characteristic (SROC) curve and calculated the area under the curve (AUC). If *P* < 0.05, the difference is statistically significant. We adopted the Begg's test and egger's test to check for publication bias, where if *p* > 0.05 this means no publication bias is found (6).

## Results

### Records Searching

**Figure 1:**
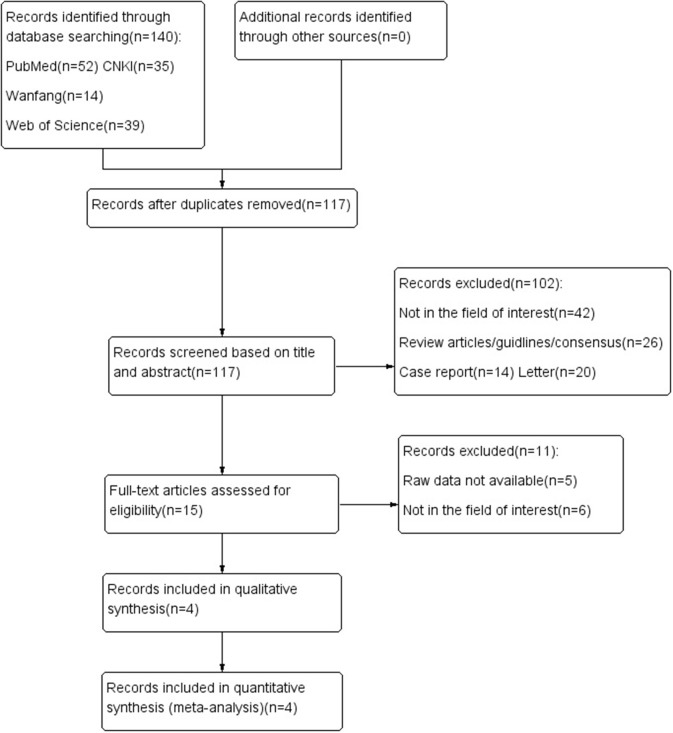
Flow diagram showing study selection process for meta-analysis.

Figure 1 shows the process of document searching and screening. A total of 140 records were originally searched. Among that, 23 records were removed because of the duplicate records; after looking through the title and abstract, 102 records were excluded. As followed as the strict inclusion and exclusion criteria, eventually 4 records were enrolled in qualitative analysis (7–10).

### Characteristics of Included Studies

The characteristics of candidate studies are shown in Table 1. All 4 investigations were retrospective study within single center. The patients were Chinese in Zhang Wei and Xia Weili's studies and non-Chinese in the other two studies. The number of the study patients ranged from 45 to 84. The mean age of the included patients ranged from 58 to 69 years. Mp-MRI was interpreted when blinded to the reference standard in all of the studies, and the results of the imaging were read by two experienced readers independently. The interval between MRI and the reference standard was not provided in these studies. Seventy out of two hundred fifty-nine included patients were female.

**Table 1:** Characteristics of included studies.

**References**	**Study period**	**Country/ Language**	**No. of patients**	**Age(yrs)**		**No. of readers**	**Golden standard**	**Research type**	**Male/female**	**Raw data**			
				**Mean**	**Range**					**TP**	**FP**	**FN**	**TN**
Zhang et al. (9)	2015.10-2017.03	China/ Chinese	55	58.67	48-68	2	Pathologic result	Retrospective study	48/7	48	8	7	157
van der Pol et al. (7)	2011.08-2016.10	USA/ English	45	69	44–89	2	Pathologic result	Retrospective study	33/12	40	9	13	63
Barchetti et al. (8)	2017.09–2018.07	Italy/ English	75	69	62–78	2	Pathologic result	Retrospective study	62/13	35	14	9	92
Xia et al. (10)	2012.10–2016-12	China/ Chinese	84	NR	NR	2	Pathologic result	Retrospective study	46/38	49	4	3	28

*TP, true positive; FP, false positive; FN, false negative; TN, true negative*.

The basic mp-MRI parameters of each study are summarized in Table 2. Zhang Wei's study used 1.5-T scanners, Christian B.'s study used both 1.5-T and 3.0-T, while the rest two studies used 3.0-T scanners. All studies used conventional sequence (T2WI) and functional imaging sequences (DWI, DCE).

**Table 2:** Characteristics of mp-MRI used by the included records.

**References**	**Scanner**		**Magnet strength (Tesla)**	**T2WI**					**DWI**					**DCE**				
	**Manufacturer**	**Model**		**TR (ms)**	**TE (ms)**	**ST (mm)**	**SG (mm)**	**FA (degree)**	**TR (ms)**	**TE (ms)**	**ST (mm)**	**SG (mm)**	**FA (degree)**	**TR (ms)**	**TE (ms)**	**ST (mm)**	**SG (mm)**	**FA (degree)**
Zhang et al. (9)	NR	Magnetom	1.5	7,000–8,000	90–102	3	1	NR	4,000	78	4	0.4	NR	180–300	1.7-4	6	2	70
van der Pol et al. (7)	Siemens /GE	NR	1.5/3.0	3,000–6,000	100–140	5	1	NR	7,800	68	6	1	NR	NR	NR	NR	NR	NR
Barchetti et al. (8)	GE	Discovery MR750W	3.0	119	119	3–4	0–0.4	90	61	61	3–4	0.3–0.	90	1.2	15	1	0	15
Xia et al. (10)	GE	SignaHDx	3.0	4,800	120	4	0.5	NR	4,000	120	5	0.5	NR	3.5	1.5	4	−2	NR

*NR, not reported; TR, repetition time; TE, echo time; ST, slice thickness; SG, slice gap; FG, flip angle*.

### Quality Assessment

**Figure 2:**
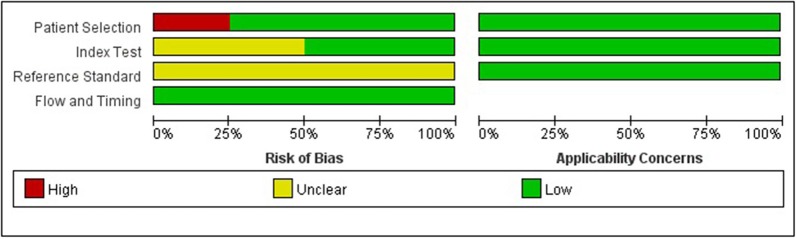
Grouped bar charts show risk of bias and applicability concerns of 4 included records using QUADAS-2.

The quality of the included studies was evaluated according to the QUADAS-2 (5), and the risk of bias and applicability concerns of 4 included studies is shown in Figure 2. In general, the quality of the included studies was considered high. Regarding the patient selection domain, Giovanni B.'s study was considered to have high risk of bias as the included patients were not identified by pathology. Regarding the index test domain, Giovanni B. and Zhang Wei's studies were considered to have unclear risk, because blinding was unclear. Regarding the reference standard domain, all 4 studies had unclear risk of bias as it is uncertain whether the interpretation of the reference standard used the blind method. Regarding the flow and timing domain, the included studies had low risk of bias. There was low concern for applicability with regard to the first three QUADAS-2 domains for all 4 included studies.

### Meta-Analysis

**Figure 3:**
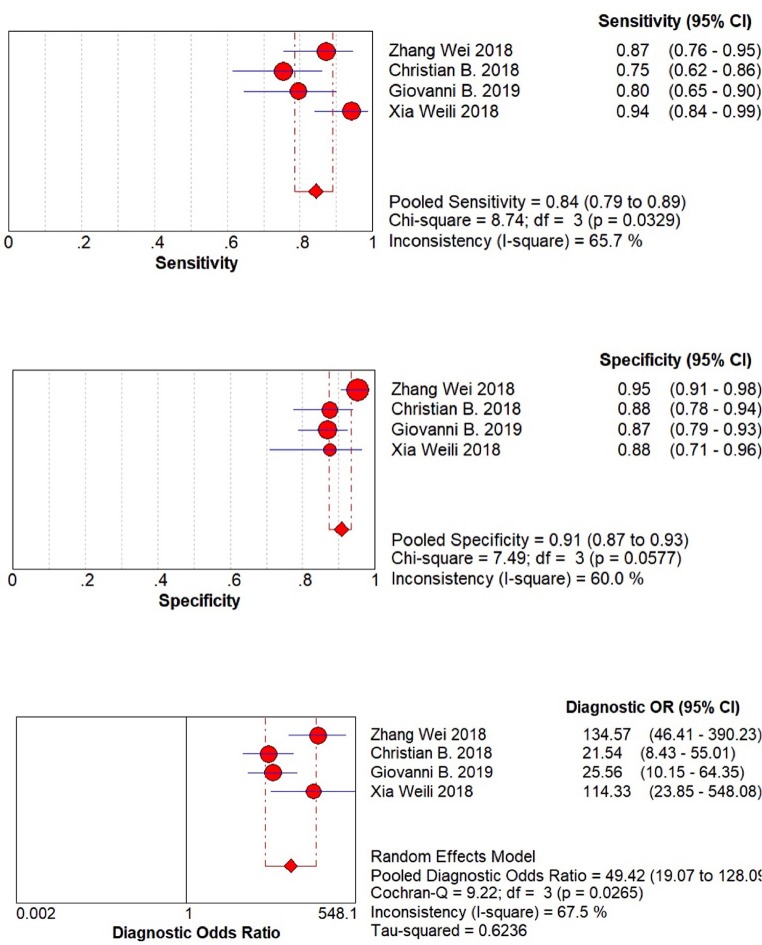
Forest plots of pooled sensitivity, specificity, and diagnostic OR.

**Figure 4:**
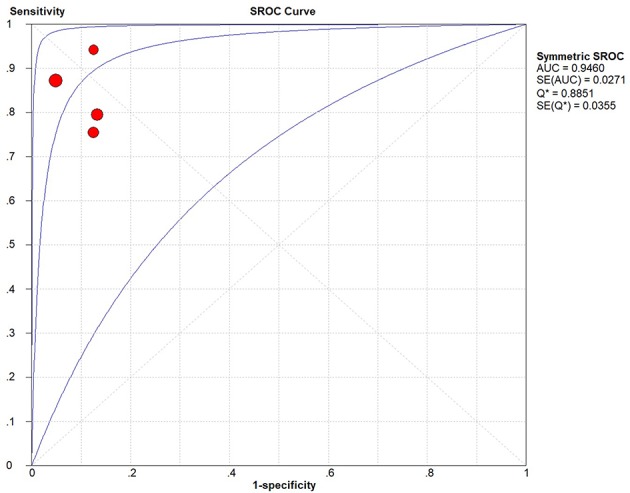
Forest plots of pooled SROC Curve.

The result of meta-analysis is showed in the Figures 3, 4. We found that the pooled sensitivity = 0.84(95% CI 0.79–0.89), pooled specificity = 0.91 (95% CI 0.87–0.93), pooled +LR = 8.24 (95% CI 4.87–13.92), pooled –LR = 0.18(95% CI 0.10–0.31), pooled DOR = 49.42(95% CI 19.07–128.09), and pooled AUC = 0.946. All these findings suggest the mp-MRI has a strong diagnostic efficiency in bladder cancer.

### Heterogeneity Test and Publication Bias

**Figure 5:**
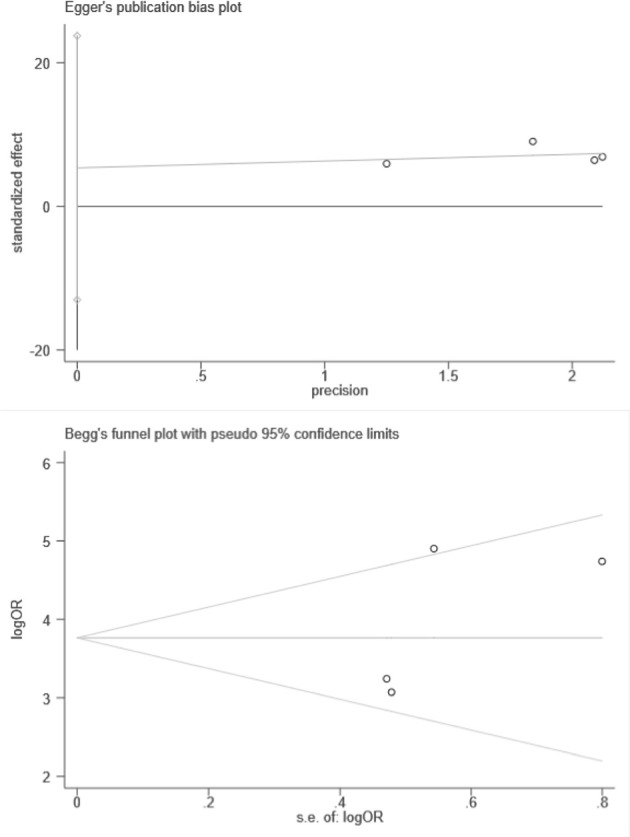
Plots of Egger's and Begg's test.

Besides, we performed Spearman correlation analysis between Sen log and (1-Spe) log, and found no threshold effect (*p* = 0.684). However, the result of heterogeneity test showed that there was a significant heterogeneity among these 4 studies within sensitivity (*I*^2^ = 65.7%), specificity (*I*^2^ = 60.0%), and diagnostic OR (*I*^2^ = 67.5%). These results indicated the randomized effects model for the above combined analysis. Furthermore, we did not find publication bias using both Begg's test (*p* = 0.497) and egger's test (*p* = 0.337), as shown in Figure 5.

## Discussion

There is an increasing number of patients who suffer from bladder cancer worldwide (1). With the development of technology and modern medicine, mp-MRI is wildly used in diagnosing and staging bladder cancer around the world (4), but its effectiveness fluctuates a lot. In this study, we integrated all current studies about mp-MRI clinical application to evaluate the diagnostic performance of mp-MRI for bladder cancer staging. The pooled sensitivity, specificity, and AUC are 0.84, 0.91, and 0.946, respectively, and these high values indicate high efficiency of mp-MRI diagnosing and staging the bladder cancer, and provide scientific evidence for clinical diagnose and decision-making.

The golden standard of diagnosis for staging of bladder cancer is the biopsy through cystoscopy, and some imaging methods such as X-ray and CT are also wildly adopted for reference. However, there are some limits of these traditional methods of diagnosis, for example, they cannot guarantee the safety and accuracy at the same time. In recent years, the MRI protocol has been wildly used in diagnosing and staging bladder cancer, and current studies demonstrate that it may largely effect the diagnostic accuracy for local staging of bladder cancer (11, 12). Especially the multi-parametric approach consists of the conventional sequence [T2-weighted anatomic imaging (T2WI)] and functional MRI techniques [dynamic contrast-enhanced (DCE) imaging and diffusion-weighted imaging (DWI)]. Compared with only using conventional sequences or only a single functional technique, the mp-MRI has a higher accuracy in diagnosing and staging bladder cancer (13). T2WI has high spatial resolution so it can differentiate the layers of the bladder. On the other hand, the functional techniques also have advantages as they can better depict the tumor itself (DWI). Furthermore, some other imaging features, such as the tumor stalk and submucosa linear enhancement, make it possible for DWI to local stage the bladder cancer (14). Therefore, combining the anatomical sequence (T2WI) with the functional sequences (DWI, DCE), the performance of MRI for diagnosing and staging bladder cancer will be improved. In addition, 3.0-T MRI benefits the tumor detection because it can increase the Signal to Noise Ratio of the image and improve the spatial and temporal resolution of the image. Multiple studies have proved that 3.0-T MRI shows a better specificity and sensitivity than 1.5-T MRI when used to diagnose and stage bladder cancer. Therefore, the guidelines recommend the use of multi-parametric 3-T MRI in the clinical diagnosis to improve the diagnostic accuracy for determining bladder cancer T stage (15). The T stage of the tumor is classified into four categories [T1 or lower, T2 (T2a or T2b), T3 (T3a or T3b), and T4 (T4a or T4b)] in accordance with the 8th TNM classification of malignant tumors which was published in 2017 (16).

On T2W images, a T2 hypo-intense band appeared by the normal detrusor muscle outlining the bladder lumen (17). Stage Ta, Tis, or T1 bladder cancer shows an intact T2 hypo-intense band. The T2 hypo-intense band and the irregular inner margin at the junction of bladder tumor and normal tissue suggest T2a stage. In addition, in the T2b stage, the T2 hypo-intense band is disrupted, without invasion of the adjacent peri-vesical fat (17, 18). When the tumor signal extends into the fat, it is considered as T3 stage, when it extends into the adjacent organs or the pelvic wall, T4 stage is considered.

On DW and DCE images, the bladder mucosa and lamina propria are enhanced, while the underlying detrusor muscle remains with high signal intensity in the early phase contrast-enhanced images about bladder cancer (17, 18). The early post-contrast images about Ta, Tis, and T1 stage show that the muscle underlying the tumor remains with high signal intensity. In addition, beneath the tumor, the enhancement of the intact submucosa linear that presents in the early phase images also indicates the stage of Ta, Tis, or T1 (11, 18, 19). On the early post-contrast images, if the inner margin at the junction of the bladder tumor and muscle is irregular, the disease is in the stage of T2a. However, in the images of stage T2b disease, the hypo-intense muscle wall is disrupted and the peri-vesical fat is not extended by the early enhancing tissue. In the images of T3 and T4 tumors, the early abnormal enhancing tissue extends into the peri-vesical fat and surrounding tissues or organs (11, 12, 19).

There are some limitations in this study. First, two of the included studies, respectively calculated the data achieved by two readers, so we had to choose one reader's results randomly. Another limitation is the small sample size, for only 4 records were included, which can influence the accuracy of the research result and make it difficult for subgroup analysis.

## Conclusion

This study supported that mp-MRI acted a high diagnostic accuracy for staging of bladder cancer, and proved that mp-MRI can be used as one of the main diagnostic methods of bladder cancer in the clinic. These findings will provide an important theoretical basis for clinical physicians to judge and make clinical decisions on bladder cancer. Also, it is necessary to conduct high-quality, multi-center studies to increase sample size to further explore the diagnostic value of mp-MRI in staging the bladder cancer.

## Author Contributions

BX put forward the idea. NZ and XW searched the literature. NZ and CW extracted and analyzed the data. SC, JW, GZ, WZ, and JL provided statistical advice. NZ wrote the manuscript. MD and MC reviewed and revised the manuscript.

### Conflict of Interest

The authors declare that the research was conducted in the absence of any commercial or financial relationships that could be construed as a potential conflict of interest.

## Abbreviations

mp-MRI: multi-parametric magnetic resonance imaging
BCa: bladder cancer
(QUADAS)-2: The Quality Assessment of Diagnostic Accuracy Studies
+LR: positive likelihood ratio
−LR: negative likelihood ratio
DOR: diagnostic odds ratio
SROC: summarized receiver operating characteristic curve
AUC: the area under the curve
CI: 95% confidence interval
T2WI: T2-weighted anatomic imaging
DCE: dynamic contrast-enhanced
DWI: diffusion-weighted imaging
TP: true positive
FP: false positive
FN: false negative
TN: true negative.
